# Application of a High Throughput Method of Biomarker Discovery to Improvement of the *Early*CDT^®^-Lung Test

**DOI:** 10.1371/journal.pone.0051002

**Published:** 2012-12-13

**Authors:** Isabel K. Macdonald, Andrea Murray, Graham F. Healey, Celine B. Parsy-Kowalska, Jared Allen, Jane McElveen, Chris Robertson, Herbert F. Sewell, Caroline J. Chapman, John F. R. Robertson

**Affiliations:** 1 Oncimmune Ltd, Nottingham City Hospital, Nottingham, United Kingdom; 2 Department of Mathematics and Statistics, University of Strathclyde, Glasgow, United Kingdom; 3 Centre of Excellence for Autoimmunity in Cancer, University of Nottingham, Nottingham, United Kingdom; Oncimmune Ltd, United Kingdom

## Abstract

**Background:**

The National Lung Screening Trial showed that CT screening for lung cancer led to a 20% reduction in mortality. However, CT screening has a number of disadvantages including low specificity. A validated autoantibody assay is available commercially (*Early*CDT®-Lung) to aid in the early detection of lung cancer and risk stratification in patients with pulmonary nodules detected by CT.

Recent advances in high throughput (HTP) cloning and expression methods have been developed into a discovery pipeline to identify biomarkers that detect autoantibodies. The aim of this study was to demonstrate the successful clinical application of this strategy to add to the *Early*CDT-Lung panel in order to improve its sensitivity and specificity (and hence positive predictive value, (PPV)).

**Methods and Findings:**

Serum from two matched independent cohorts of lung cancer patients were used (n = 100 and n = 165). Sixty nine proteins were initially screened on an abridged HTP version of the autoantibody ELISA using protein prepared on small scale by a HTP expression and purification screen. Promising leads were produced in shake flask culture and tested on the full assay. These results were analyzed in combination with those from the *Early*CDT-Lung panel in order to provide a set of re-optimized cut-offs. Five proteins that still displayed cancer/normal differentiation were tested for reproducibility and validation on a second batch of protein and a separate patient cohort. Addition of these proteins resulted in an improvement in the sensitivity and specificity of the test from 38% and 86% to 49% and 93% respectively (PPV improvement from 1 in 16 to 1 in 7).

**Conclusion:**

This is a practical example of the value of investing resources to develop a HTP technology. Such technology may lead to improvement in the clinical utility of the *Early*CDT­-Lung test, and so further aid the early detection of lung cancer.

## Introduction

The role of the immune system in carcinogenesis remains incompletely understood despite decades of research. It is known, however that a patient may display a specific host immune response to tumor cells and that this may have implications for tumor progression [1]. Humoral responses to cancer-associated antigens are well documented [2] and detecting autoantibodies (AAb) could lead to new insights into this process of carcinogenesis as well as provide biomarkers for early detection of cancer and subsequent patient management.

It is well accepted that early detection (at a curable stage) of most types of cancer will lead to decreased mortality. For instance the recently published findings of the National Lung Screening Trial (NLST) in the USA showed that early detection of lung cancer using low dose spiral computed tomography (CT) screening led to a 20% reduction in lung cancer mortality [3]. However, CT screening for lung cancer has a number of potential difficulties including cost and the number of false positive tests, both of which are linked to the low specificity associated with this imaging test [4], [5]. Hence the search for improved biomarkers that could be used in combination with imaging for cancer screening remains an important goal.

In order to be clinically useful, biomarker assays need to be highly robust and reproducible with levels of clinical sensitivity and specificity appropriate for the particular application. Such techniques must undergo quality assurance during development, and in subsequent laboratory and clinical use, to ensure the accuracy of the reported result. The *Early*CDT-Lung test [6]–[9] meets these criteria and is currently used in the USA, to aid early detection of lung cancer. *Early*CDT-Lung took over 7 years to develop, validate (technically and clinically) and commercialize. Development of the panel involved individually cloning antigens and then screening them for their ability to differentiate between patients with lung cancer and high risk individuals with no evidence of malignant disease who had been matched for age, gender and smoking status [7]. The test has now been available for three years and audit of its clinical performance has shown it to be exactly as predicted from validation studies [9], [10]. Having confirmed proof of principle for the use of AAb panels for early detection of lung cancer, similar tests for other cancer types (e.g. breast, colon, liver), will now be developed. However, this will require a more efficient method of lead discovery in order to accelerate development timelines and reduce costs.

Techniques such as SEREX [11] and SERPA [12] have been used for the identification of biomarker leads for AAb assays. However, the detection methods used in such discovery systems are often technically challenging and the lack of publications describing AAb assays in routine use in the clinical setting would suggest that technology transfer from discovery systems to robust assays while maintaining clinical utility and performance is rarely successful.

A High Throughput (HTP) cloning and expression system has recently been described [13] which utilizes ligation-independent cloning (LIC) [14] and is performed in a microtiter plate format allowing cloning, expression and purification of up to 96 recombinant proteins at one time and in a matter of weeks. LIC does not require exogenous ligase, resulting in high cloning efficiency (generally>80% success). Micro-scale metal chelate affinity matrices in 96-well plate format allow purification and simultaneous on-column refolding of expressed proteins with yields generally in the range of 1–2 mg purified product. Proteins expressed and purified using this method, then used as capture antigens in the detection of AAb in lung cancer patients by ELISA have been demonstrated to compare well with recombinant proteins expressed and purified by larger scale standard methods [13]. Since the screening assay is an ELISA, the results are directly transferrable to other robust ELISA based technologies such as the *Early*CDT-Lung test. The HTP version of the AAb ELISA utilizes a 2 point assay (compared to a 5 point assay for the *Early*CDT-Lung test) so enabling the analysis of 21 proteins (compared to 7 for the 5 point assay) per assay, allowing a more economical use of valuable and limited patient sera. The HTP system is therefore an ideal method for the development of panels of antigens for the detection of cancers in addition to lung and also for identification of new biomarkers that will lead to an improvement in the clinical performance of the *Early*CDT-Lung test as described here. The aim of this study was to demonstrate the successful clinical application of this strategy to identify biomarkers which when added to the *Early*CDT-Lung panel improve its sensitivity and specificity (and hence positive predictive value, (PPV)).

## Materials and Methods

### Ethics Statement

All control samples used in this study were collected by the University of Nottingham within the East Midlands area of the UK through recruitment drives at various public and private places. Participants all gave their full written informed consent and approval from the University of Nottingham Medical Research Ethics Committee (Ethics Reference Number BT/07/2007) was also gained.

All patient samples were purchased from Kiev Biopharma, Asterand, Sera Lab or Indivumed (Table S1). They were recruited by medical practitioners at treatment sites, who obtained full written informed consent. Blood samples were obtained after diagnosis but before receiving any anti-cancer treatment.

### Patient samples

Two separate cohorts of patients with all stages and class of newly diagnosed lung cancer were identified (Table S2). Cohort 1 contained 165 lung cancer patients (median age 62; range 34–87) and 165 controls (62; 34–87) while cohort 2 had 100 lung cancers (64; 23–87) and 100 controls (64; 23–87), respectively.. Patients and control individuals were predominantly high risk individuals i.e. long standing smokers, all with no history of malignant disease. All patients with lung cancer were, as far as possible, individually matched predominantly by gender and age, and then smoking history, to a control individual (Table S1).

### HTP cloning (HTPC)

HTP cloning using LIC compatible *E.Coli* protein expression vectors was performed as described by Macdonald *et al* [13]. Briefly, the linear LIC vectors and human TAA DNA sequences (amplified by PCR using IMAGE clone templates, (Geneservice)) were appropriately T4 treated, annealed and introduced into *E.Coli*. Transformation cultures were grown on Luria Bertani agar, two colonies were picked for each LIC construct and plasmid DNA was prepared (CosMC kit, Agencourt). Both clones were analyzed by insert specific PCR for an insert of the correct size by agarose gel electrophoresis. Construct identification was verified by DNA sequencing (Source Bioscience). Constructs denoted with NLIC or CLIC were HTP cloned into the NLIC and CLIC vectors respectively, expressing N-BirA or C-BirA fusion proteins where the TAA was fused to the BirA and His tags by a Glycine Serine linker at the N or C termini respectively. All other constructs were cloned by restriction site cloning into pET21b (Novagen, Merck) with a His tag at the C terminus. When BirA was cloned into the construct it was at the N termini and noted in the construct name as BirA.

### HTP Expression (HTPE) & Purification

The HTP expression and purification was performed as described by Macdonald *et al* [13]. Briefly, TAA containing LIC constructs and previously cloned TAA pET21b vectors (106 constructs in all) were expressed in *E.Coli* in 30 ml cultures. Up to 2 mg of pure protein was produced for 69 of these proteins. The reduced protein samples were analyzed for purity, yield and molecular weight by SDS-PAGE and concentrations were estimated by comparison to bovine serum albumin standards.

Promising leads were scaled up to 200 ml shake flask expression cultures (R&D batches). Cell pellets were lyzed and purified as previously described [15]. Proteins were analyzed for molecular weight and purity using SDS-PAGE and Western Blotting and quantified by Bradford assay (Biorad).

### Assays

#### Abridged HTP Assay

The 2 point abridged HTP (HTPA) version of the full assay was performed as described earlier [13] to test 69 fusion proteins. The Kolmogorov-Smirnov (K-S) test was used to quantify the distance between the empirical distribution functions of the signal from cancer and normal populations for each fusion protein. K-S scores were plotted as histograms for both 100 nM and 50 nM plate coating concentrations. Scatter plots were also used to visually compare the mean OD signal between cancer and normal populations.

#### Research and Development (R&D) assay

The validated AAb assay technique, using a 5 point titration of each individual antigen (R&D batches of any lead antigen), was performed as previously described [6], [7] with each antigen concentration being tested in duplicate. For assays involving lead antigens, no calibration was applied, so in order to minimize assay variability that may bias results, matched cancer and normal samples were assayed on the same day. K-S scores for the comparison of cancer and normal populations were calculated for 160 nM and 50 nM coating concentrations and plotted as histograms. Samples were judged to be positive for the presence of a specific AAb when the ELISA OD mean was above a cut-off for the corresponding antigen. Cut-offs were chosen individually for each antigen in order to provide the maximum number of cancers with positive signal (sensitivity) and the controls with negative signal (specificity).

#### R&D *Early*CDT-Lung

This was performed as described previously [6], [7] using the commercial panel of antigens and applying calibration to provide a results in reference units (RU) following manual data cleaning. The cut-off values used in the present commercial test were used when comparing the performance of the *Early*CDT-Lung Test with that of any modified panel. However, when sensitivity and specificity values were calculated for panels modified by the inclusion of one or more lead antigens, the cut-offs were re-optimized in order to provide optimal sensitivity and specificity for the modified AAb panel as a whole. When these re-optimized cut-offs were applied to the *Early*CDT-Lung antigens the panel was described as the *e*CDT-Lung R&D panel to discriminate it from the commercial test with commercial cut-offs applied. All commercial *Early*CDT-Lung panel antigens have N terminal BirA tags and C terminal His tags.

## Results

### Discovery of new TAAs for *Early*-CDT Lung Assay by HTPA

**Figure 1:**
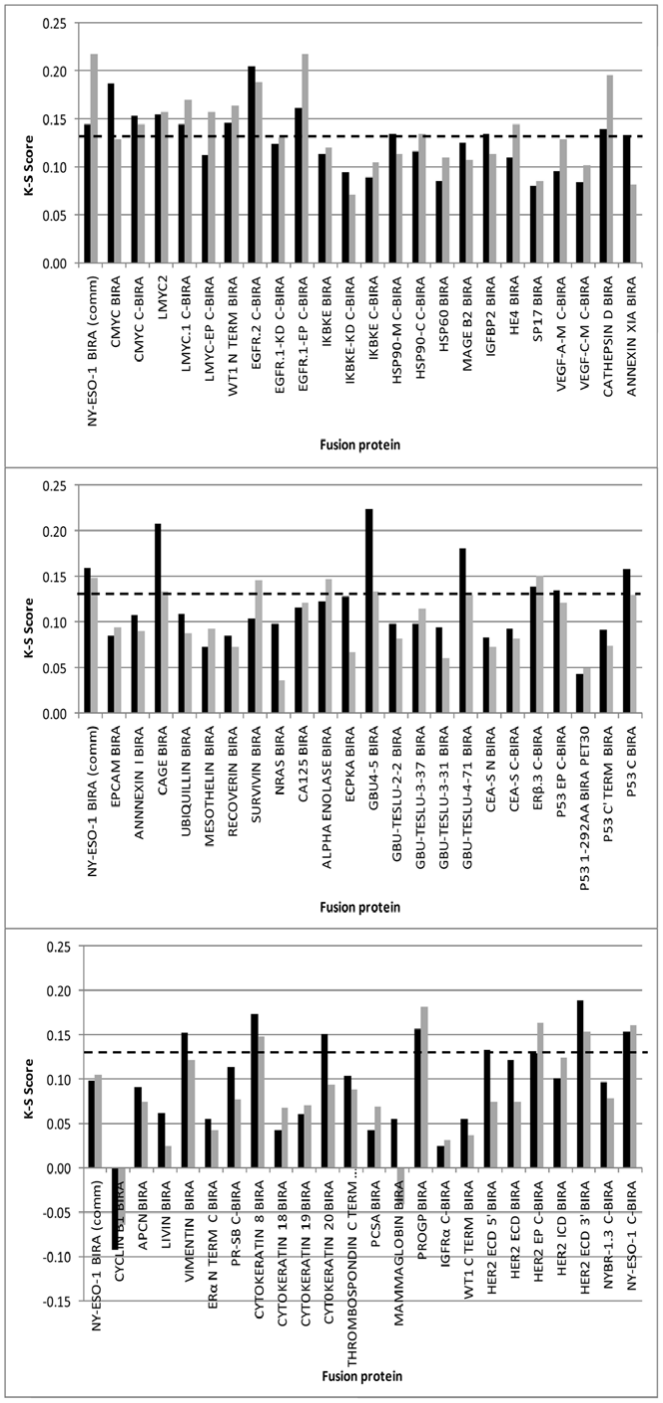
K-S Score for HTPA analysis of 69 fusion proteins. Calculations were based on populations of 165 lung cancers and 165 individuals with no evidence of malignant disease (cohort 1). Bars represent fusion protein plated at 100 nM (black) and 50 nM (grey). NY-ESO-1 BirA (comm) = the cancer antigen control.

**Figure 2:**
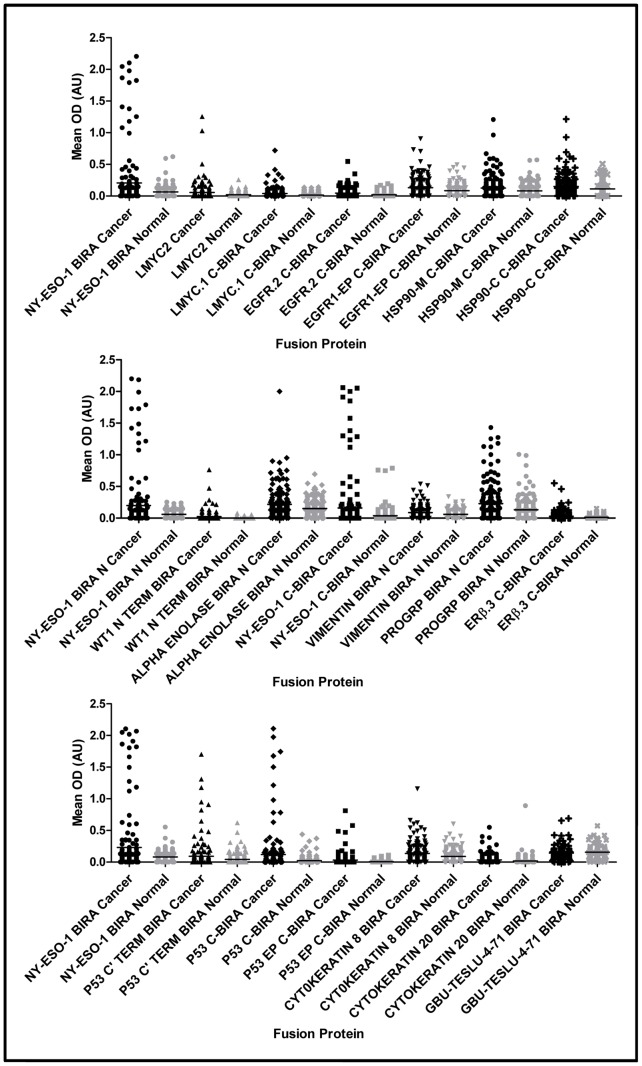
Cancer : normal differentiation in HTPA. Scatter plots showing level of signal from autoantibody binding to fusion proteins coated at 100 nM in 165 cancer patients (black) and 165 individuals with no evidence of malignancy (grey). NY-ESO-1 BirA = the cancer antigen control.

HTPA lung assays were run, to test 69 fusion protein batches, on cohort 1. K-S Scores were calculated and are shown in Figure 1. It can be seen that 29 fusion proteins gave K-S scores above an arbitrary cut-off of 0.13 at either 100 or 50 nM coating concentration. The mean OD data (by antigen) for each of the 330 serum samples were plotted, according to disease status on scatter plots and those proteins with K-S scores of greater than 0.13 and which also showed cancer normal differentiation on scatter plots (Figure 2) were termed ‘leads’ and taken forward for further analysis. These 18 leads were expressed in shake flask culture (‘R&D’ batch) and after purification and characterization the fusion proteins were tested on the 5 point R&D assay.

### Lung lead confirmation and validation results

**Figure 3:**
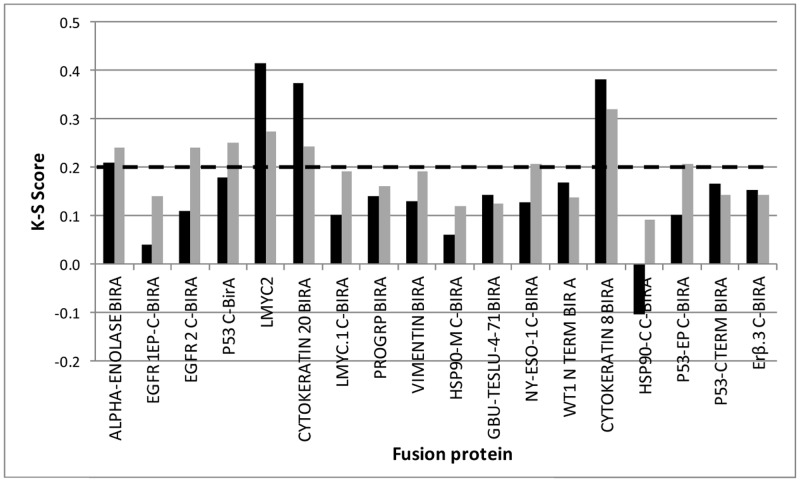
K-S Score for R&D assay analysis of scaled up fusion proteins. Fusion proteins shown by HTPA to be potential leads were expressed in shake flask culture, purified and used as capture antigens for measurement of AAb by 5 point R&D assay. Calculations were based on populations of 100 lung cancers and 100 individuals with no evidence of malignant disease (cohort 2). Bars represent fusion protein plated at 160 nM (black) and 50 nM (grey).

An R&D batch of each lead was prepared and employed as capture antigen in the R&D assay. The performance of these batches along with the *Early*CDT panel antigens [9] was assessed using Cohort 2 samples. K-S Scores were calculated for the lead antigens in this data set and are shown in Figure 3. Eight fusion proteins gave K-S scores greater than or equal to a more stringent cut-off of 0.2. It was considered that in order to gain the best assay performance from an AAb panel extended by the inclusion of lead proteins, the cut-offs of all antigens in the panel would need to be re-set. To this end, cut-offs were chosen individually for each of these eight antigens and simultaneously, for the antigens in the *Early*CDT-Lung R&D panel in order to provide the maximum number of cancers with positive signal and controls with negative signal. This was termed the *e*CDT-Lung R&D panel.

**Figure 4:**
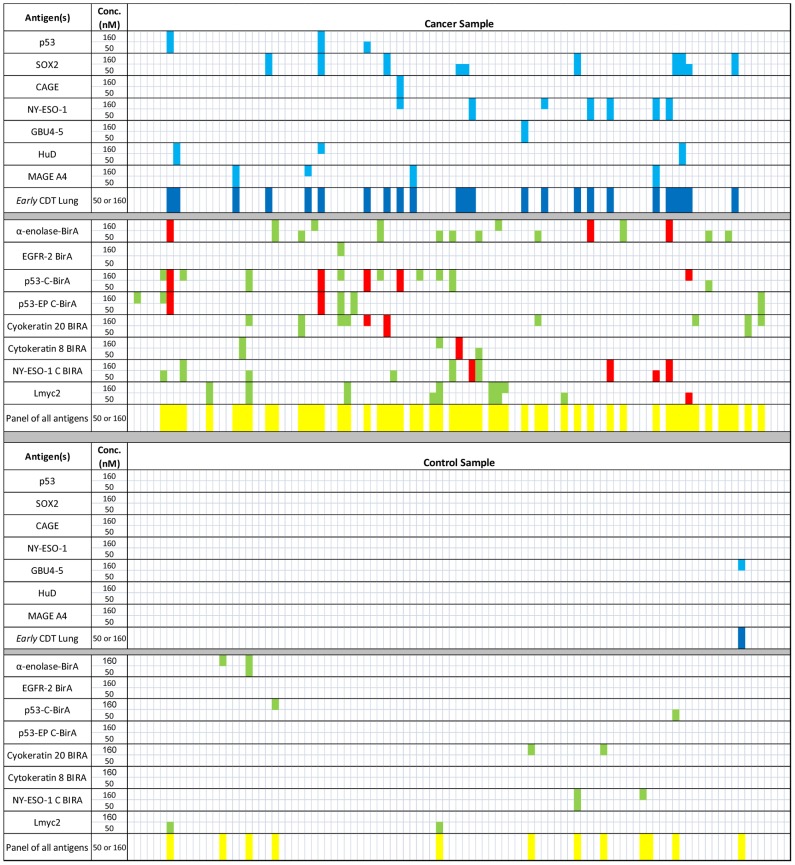
Panel positivity of scaled up lung cancer leads. Fusion proteins shown by HTPA to be potential leads were expressed in shake flask culture, purified and used as capture antigens for measurement of AAb by 5 point R&D assay. Cut-offs were optimized to give maximal cancer normal differentiation. Samples that were positive for the *e*CDT-Lung R&D panel antigens are shown as light blue lines with *e*CDT-Lung R&D panel positivity shown as dark blue lines. Positivity of lead antigens is shown as red lines if that sample was already positive for one of the antigens in the *e*CDT-Lung R&D panel (non-additive) and green lines if the lead identified a positive not found using *e*CDT-Lung R&D panel (additive). Positivity of the total panel of 15 antigens is shown as yellow lines.

These optimized cut-offs were used to generate bar charts to demonstrate the AAb positivity of the 8 lead fusion proteins with K-S scores greater than 0.2 compared with the *e*CDT-Lung R&D panel for Cohort 2 (Figure 4). It can be seen that on this particular cohort, composed predominantly of non-small cell lung cancers, the *e*CDT-Lung R&D assay panel alone gave a sensitivity of 24% and a specificity of 99% using cut-offs that had been re-optimized in combination with the lung leads identified by HTPA. Samples identified as positive for any lead were separated into two categories: ‘non-additive positives’ if also positive to one of the original seven *e*CDT-Lung R&D assay panel antigens and indicated by red bars; and ‘additive positives’ if not previously identified by *e*CDT-Lung R&D assay and indicated by green bars (Figure 4). The antigens having the most additive positives were those that had the greatest potential to increase the performance of the *e*CDT-Lung R&D panel as a whole as opposed to those with the highest individual sensitivity and specificity. Inclusion of all 15 antigens (seven *e*CDT-Lung R&D panel antigens plus eight leads) provided panel sensitivity of 53%, however the specificity performance of this extended panel dropped from 96% to 88%. Therefore it was essential to also consider the individual performance of leads in the context of positivity amongst normal individuals and the effect on panel specificity.

Table 1 shows the positivity of leads in cancer and normal groups compared with the *e*CDT-Lung R&D panel. It can be seen that although the K-S score calculated for EGFR2 C-BirA (Isoform 2) was relatively high (Figure 3) the maximum cancer normal differentiation that could be achieved using optimized cut-offs was small. Cytokeratin 8 BirA, p53-EP C-BirA and NY-ESO-1 C-BirA also provided a modest improvement in cancer normal differentiation over the *e*CDT-Lung R&D panel. Since very similar antigens to the latter two are already present in the *e*CDT-Lung R&D panel (p53 BirA and NY-ESO-1 BirA), these were not pursued further. However, cytokeratin 8 is a molecule of different biochemical nature to the other antigens in the commercial panel and since the specificity of this antigen was high it was decided to take it forward to the validation stage. Therefore, five antigens (alpha enolase BirA, p53 C-BirA, cytokeratin 8 BirA, cytokeratin 20 BirA, and Lmyc2 (isoform 2 without a BirA tag)) underwent further evaluation on a separate cohort. The antigens were termed confirmed leads and their sensitivity and specificity was validated on a separate sample set (cohort 1) by applying the same optimized cut-offs that had been derived using cohort 2.

**Table 1:** Positivity of scaled up lung cancer leads compared with the *Early*CDT-Lung panel.

Fusion Protein	Overall positivity		Additive positivity		Net additive gain
	Cancers	Controls	Cancers	Controls	Cancers - controls
***Alpha enolase BirA***	***15%***	***2%***	***12%***	***2%***	***10%***
EGFR 2 C-BirA	1%	0%	1%	0%	1%
***p53 C-BirA***	***14%***	***2%***	***9%***	***2%***	***7%***
p53-EP C-BirA	7%	0%	5%	0%	5%
***Cytokeratin 20 BirA***	***10%***	***2%***	***8%***	***2%***	***6%***
***Cytokeratin 8 BirA***	***4%***	***0%***	***3%***	***0%***	***3%***
NY-ESO-1 C-BirA	10%	2%	6%	2%	4%
***Lmyc2***	***10%***	***2%***	***9%***	***2%***	***7%***

Cut-offs were optimized to give the maximum number of positive cancers and negative control samples for both the leads and the *e*CDT-Lung R&D panel antigens. Overall positivity in cancers and controls is given for each lead as well as the additive positivity over the *e*CDT-Lung R&D panel. Antigens defined as confirmed leads and taken forward for validation on a separate sample cohort are shown in bold italic font.

From the 18 leads identified by HTPA, the 5 antigens detailed above were taken forward for further analysis by R&D assay on scaled up R&D protein batches (highlighted in bold, italic font in Table 1). The optimized cut-off values determined using sample cohort 2 were validated by applying them to R&D assay results from cohort 1. The new cut-offs derived for the *e*CDT-Lung R&D assay panel were also applied to the commercial antigens. The cohort 1 R&D assay sensitivity and specificity values for each individual antigen are given in Table 2. It can be seen that the *e*CDT-Lung R&D test alone gave a panel sensitivity and specificity of 36% and 93% respectively when tested on this patient cohort. The confirmed leads alone gave sensitivity and specificity values ranging from 5 to 9% and 98 to 100% respectively. The sensitivity values for *e*CDT-Lung R&D panel in combination with each of the confirmed leads individually is highlighted in italic font and it can be seen that addition of each of the leads led to an improvement in sensitivity of the panel but did not always result in a decrease in specificity. Table 2 shows data for every combination of 2 and 3 confirmed leads with the *e*CDT-Lung R&D panel. The best combination of three confirmed leads with the panel was with either Lmyc2 and cytokeratin 20 or alpha-enolase and cytokeratin 20 which both led to sensitivity of 46% and specificity of 92%.

**Table 2:** Sensitivity and specificity of confirmed leads.

AAb Panel	*e*CDT-Lung R&D Assay		Apha enolase BirA		p53 C-BirA		Lmyc2		Cytokeratin 20 BirA		Cytokeratin 8 BirA	
	Sens	Spec	Sens	Spec	Sens	Spec	Sens	Spec	Sens	Spec	Sens	Spec
Antigen / Panel Alone	35.8%	93.3%	**9%**	**99%**	**9%**	**98%**	**7%**	**99%**	**6%**	**99%**	**5%**	**100%**
*e*CDT-Lung R&D Assay	N/A	N/A	*39.4%*	*92.1%*	*36.4%*	*92.7%*	*39.4%*	*92.1%*	*40.0%*	*93.3%*	*39.4%*	*93.3%*
*e*CDT-Lung R&D Assay+alpha enolase BirA			N/A	N/A	43.0%	91.5%	46.1%	90.9%	46.1%	92.1%	43.0%	92.1%
*e*CDT-Lung R&D Assay+p53 C-BirA					N/A	N/A	40.0%	91.5%	40.6%	92.7%	39.4%	92.7%
*e*CDT-Lung R&D Assay+Lmyc2							N/A	N/A	43.6%	92.1%	42.4%	92.1%
*e*CDT-Lung R&D Assay+cytokeratin 20 BirA (CK 20)									N/A	N/A	43.0%	93.3%
*e*CDT-Lung R&D Assay+cytokeratin 8 BirA (CK 8)											N/A	N/A
*e*CDT-Lung R&D Assay+alpha enolase+p53 C-BirA					N/A	N/A	43.6%	90.3%	43.6%	91.5%	43.0%	92.1%
*e*CDT-Lung R&D Assay+alpha enolase+Lmyc2							N/A	N/A	46.7%	90.9%	42.5%	91.5%
*e*CDT-Lung R&D Assay+alpha enolase+CK 20									N/A	N/A	46.1%	92.1%
*e*CDT-Lung R&D Assay+alpha enolase+CK 8											N/A	N/A
*e*CDT-Lung R&D Assay+p53 C-BirA+Lmyc2							N/A	N/A	44.2%	91.5%	42.5%	91.5%
*e*CDT-Lung R&D Assay+p53 C-BirA+CK 20									N/A	N/A	43.0%	92.7%
*e*CDT-Lung R&D Assay+p53 C-BirA+CK 8											N/A	N/A
*e*CDT-Lung R&D Assay+Lmyc2+CK 20									N/A	N/A	46.1%	92.1%
*e*CDT-Lung R&D Assay+Lmyc2+CK 8											N/A	N/A

Cut-offs that had been optimised on the cohort 2 sample set using R&D batches of protein were applied to the results of assays of the cohort 1 samples to validate sensitivity (sens) and specificity (spec) for each confirmed lead alone (bold) and in combination with the *e*CDT-Lung R&D panel (italic). Sensitivity and specificity values are also given for every combination of two or three confirmed leads in when added to the *e*CDT-Lung R&D panel.

Table 3 summarizes and compares the results from cohort 2 which was used to determine optimized cut-offs and cohort 1. It can be seen that although the two cohorts varied in their level of sensitivity and specificity for both the *Early*CDT-Lung test (with commercial cut-offs applied) and *e*CDT-Lung R&D panel test alone, when the commercial panel was combined with the confirmed leads, results between the 2 cohorts were very similar. In both cases, the addition of four antigens to the panel resulted in specificity values in excess of 90% while sensitivity was improved to around 50%.

**Table 3:** Summary and Comparison of Panel Performance for Cohorts 1 and 2.

*Early*CDT-Lung Panel or *e*CDT-Lung R&D Panel	Lead Antigens from HTP	Cohort 1		Cohort 2	
		Specificity	Sensitivity	Specificity	Sensitivity
***All (commercial cut-offs)***	***None***	***86%***	***38%***	***96%***	***30%***
All (optimized cut-offs)	None	93%	36%	99%	24%
All (optimized cut-offs)	All	90%	49%	91%	51%
All (optimized cut-offs)	p53 C-BirA omitted	91%	49%	93%	48%
p53 BirA omitted	All	90%	49%	91%	51%
GBU4-5 BirA omitted	All	92%	49%	92%	50%
GBU4-5 BirA omitted	p53 C-BirA omitted	93%	49%	94%	47%
p53 BirA & GBU4-5 BirA omitted	All	93%	49%	92%	50%

Data derived from application of the commercial panel cut-offs to the *Early*CDT-Lung panel are shown in bold, italic font. Cut-offs were then optimised on cohort 2 and then applied to the results obtained from cohort 1. The effect of dropping one or more antigens from the panel is shown.

## Discussion

The HTP method of lead discovery has successfully identified five antigens that have potential to improve the performance of the *Early*CDT-Lung test for measurement of AAbs for the early detection of lung cancer. However further optimization and technical and clinical validation is required before the antigens can be used in the clinical setting. This will include development of a calibration system for each antigen and optimization of the cut-off of each antigen in the final commercial panel by Monte Carlo analysis [7], [16] in order to maximize the performance of the panel as a whole. Large scale commercial batch production will also need to be developed and optimized and assay performance verified.

The K-S score is a recognized statistical test and has been used here as a preliminary measure of the difference in signals between cancer and control populations. However, as was shown in the case of EGFR2 C-BirA, a relatively high K-S score (Figure 3) does not always lead to high cancer normal differentiation (Table 1) since it tends to focus on the middle of the data distributions. This demonstrates the need for a second method of lead evaluation such as assessment of individual sensitivity and specificity values (Tables 1 and 2) which focuses on the upper end of the distributions.

This study has also demonstrated that finding new proteins to improve a panel that already contains a broad range of antigens that provide robust cancer/normal differentiation is not straightforward or easily achieved. The best leads identified provided real but modest improvements in the performance of the *Early*CDT-Lung test. Therefore while additional antigens will be explored, alternative ways of improving the assay performance must also be pursued. These include adopting a within subject approach by taking serial measurements from an individual and looking for changes from their own baseline value rather than waiting until the AAb assay result crosses a threshold defined by a high risk population in what is effectively a between-subjects comparison.

The data presented here would show that improvement in the sensitivity and specificity from 38% and 86% (as in the commercial *Early*CDT-Lung test performance on cohort 1) to 49% and 93% respectively (when the confirmed leads were added and cut-offs re-optimized), would result in an improvement in PPV from 1 in 16 to 1 in 7 (based on a prevalence of 2.4%). A test with a PPV of 1 in 7 will result in the recall of approximately 7 patients for further testing in order to detect 1 patient with lung cancer. This is compared with low dose spiral CT which has a PPV of 1 in 36 due to its low specificity for distinguishing malignant from non-malignant nodules (Swenson et al 2005). Follow up of a positive CT scan may include biopsy and further imaging such as positron emission tomography (PET) scan which have cost and morbidity implications.

De novo methods of biomarker discovery such as SEREX [11] and SERPA [12] have the ability to probe extremely large repertoires of molecules. However, the main disadvantages of such methods are in the difficulties they pose in technology transfer. For instance discovery of a lead does not guarantee that the lead can be produced in amounts high enough to be commercially viable. In addition such discovery methods often employ detection technologies that are too technically demanding or not sufficiently reproducible to transfer into the routine clinical setting. The HTP method of lead discovery described here requires a prior knowledge of the proteins to be screened, which can be obtained from the current literature since new potential targets are being described virtually every day. However it has a distinct advantage over proteomic and genomic screening strategies, in that the screened proteins are produced recombinantly in *E. Coli* with expression optimization being an integral part of the screening process [13]. Commercial scale production should therefore be relatively easy to achieve through technology transfer to large scale production and purification methodologies. In addition, the screening test is an ELISA which is a tried and tested, robust method used routinely in clinical chemistry laboratories. Both of these characteristics lend themselves well to technology transfer to the clinic.

This study identified five new antigens that have the potential to improve the performance of the *Early*CDT-Lung test for the early detection of lung cancer. Alpha enolase is a member of a family of glycolytic enzymes expressed in most tissues. It has two forms; alpha enolase (48 kDa) and Myc-binding protein-1 (MGP1, 37 kDa), which down regulates the activity of the c-myc protooncogene [17]). Alpha enolase has been identified as an autoantigen in a number of infectious and autoimmune diseases such as Hashimoto's encephalopathy [18], Behcet's disease [19] and severe asthma [20]. Cytokeratin 8 has been identified as a plasminogen-binding protein expressed on the external surfaces of hepatocytes and breast carcinoma cells [21]. Antibodies to cytokeratin 8 (e.g. CAM 5.2) can be used to differentiate lobular from ductal carcinoma of the breast [22]. Cytokeratin 8 is often used together with cytokeratins 18 and 19 to differentiate cells of epithelial origin from hematopoietic cells in tests that measure circulating tumor cells in blood [23]. Cytokeratin 20 is another member of the family of keratin cytoskeletal proteins. It is encoded by the *KRT20* gene [24] and is a major cellular protein of mature erythrocytes and goblet cells as well as being found specifically in the gastric and intestinal mucosa [25]. The expression spectrum of cytokeratin 20 in carcinomas resembles that observed in the corresponding normal epithelia of origin. Immunohistochemistry studies have shown positivity to this protein in adenocarcinomas of the colon, mucinous ovarian tumors, transitional-cell and Merkel-cell carcinomas and frequently also in adenocarcinomas of the stomach, bile system, and pancreas. However, most squamous cell carcinomas and most adenocarcinomas from other sites such as breast and interestingly, lung, are essentially negative [26]. Lmyc is a member of the myc family of proto oncogenes that are found to be aberrantly expressed in a variety of tumors. It was first discovered through its homology with the transforming gene (v-myc) of the avian myelocytomatosis virus MC29 in the amplified sequences of a small cell lung tumor [27]. In normal tissues, expression of Lmyc is restricted to embryonic development and a few adult tissues. Lmyc protein is a phosphoprotein that has a very short biological half-life and binds DNA. It is believed to have a role in transcriptional regulation and regulation of cellular proliferation [28]. Lmyc isoform 1 is a 364 amino acid protein of which isoform 2 is a truncated 207 residue version that shares the first 166 amino acids and terminates in a differing 41 amino acid sequence. Both isoforms are believed to be functional. Isoform 2 is thought to be expressed in human adult normal testis and at low levels in lung adenocarcinomas [29].

The construct based on p53 (p53 C-BirA) also performed well with respect to individual sensitivity and specificity. However, it did not consistently improve the assay performance in both independent sample sets. This may be due to the fact that this antigen is already in the test panel, albeit in a slightly different construct (p53 BirA, restriction site cloned using pET21b). This demonstrates the need to include antigens of different biochemical nature in panels for the measurement of autoantibodies in order to achieve the highest sensitivity and specificity for a panel assay.

## Summary

The HTP method of biomarker discovery described here has identified promising leads that are expected, after further optimization and technical and clinical validation, to improve the performance of the current *Early*CDT-Lung panel. It is envisaged that this approach will be applicable to other cancers and subtypes. This new HTP discovery pipeline provides a more rapid assessment of newly identified biomarkers from the literature in a very cost and time efficient manner, to ultimately aid early detection. The ultimate aim is to combine AAb tests with established screening strategies (such as low dose spiral CT in lung cancer) in order to identify patients at extremely high risk of having malignant disease, thus sparing more patients the trauma of a false positive diagnosis and the ensuing follow-up procedures. Defining optimum screening methods in different populations will save lives and ensure more effective use of limited resources in the national healthcare organizations.

## Supporting Information

Table S1 Break down of cohorts by collection centre and smoking history. Cohort 1 contained 165 lung cancer patients and 165 controls while cohort 2 had 100 lung cancers and 100 controls. All patients with lung cancer were, as far as possible, individually matched predominantly by gender and age, and then smoking history, to a control individual.
(DOCX)

Table S2 Breakdown of cohorts by cancer stage and disease class. Determined according to WHO criteria with patient numbers per cohort quoted.
(DOCX)
