# Supplementary material for: Application of a High Throughput Method of Biomarker Discovery to Improvement of the EarlyCDT®-Lung Test
Source: PLoS One. 2012 Dec 13;7(12):e51002. doi: 10.1371/journal.pone.0051002 (PMC3521770; doi:10.1371/journal.pone.0051002)
Supplement: Table S1 — Break down of cohorts by collection centre and smoking history. Cohort 1 contained 165 lung cancer patients and 165 controls while cohort 2 had 100 lung cancers and 100 controls. All patients with lung cancer were, as far as possible, individually matched predominantly by gender and age, and then smoking history, to a control individual. (DOCX) [file pone.0051002.s001.docx]

|  |  | **Cohort 1** | | **Cohort 2** | | |  |
| --- | --- | --- | --- | --- | --- | --- | --- |
|  |  | **Cancers** | **Controls** | | **Cancers** | **Controls** | |
|  |  |  |  | |  |  | |
| **Collection Centre** | Kiev Biopharma | 106 | 0 | | 68 | 0 | |
|  | Indivumed | 59 | 0 | | 0 | 0 | |
|  | Asterand | 0 | 0 | | 30 | 0 | |
|  | Sera Lab | 0 | 0 | | 2 | 0 | |
|  | UoN* | 0 | 165 | | 0 | 100 | |
|  |  |  |  | |  |  | |
| **Smoking History** | Current | 72 | 35 | | 51 | 8 | |
|  | Ex | 68 | 98 | | 30 | 47 | |
|  | Never | 25 | 32 | | 16 | 45 | |
|  | Unknown | 0 | 0 | | 3 | 0 | |

*University of Nottingham
