# Supplementary material for: Application of a High Throughput Method of Biomarker Discovery to Improvement of the EarlyCDT®-Lung Test
Source: PLoS One. 2012 Dec 13;7(12):e51002. doi: 10.1371/journal.pone.0051002 (PMC3521770; doi:10.1371/journal.pone.0051002)
Supplement: Table S2 — Breakdown of cohorts by cancer stage and disease class. Determined according to WHO criteria with patient numbers per cohort quoted. (DOCX) [file pone.0051002.s002.docx]

|  |  | **Cohort 1** | **Cohort 2** |
| --- | --- | --- | --- |
|  |  |  |  |
| **Cancer Staging** | Stage I | 56 | 59 |
|  | Stage II | 15 | 12 |
|  | Stage III | 25 | 23 |
|  | Stage IV | 6 | 2 |
|  | Stage III/IV | 29 | 0 |
|  | Unknown | 34 | 4 |
|  |  |  |  |
| **Disease Class** | SCLC* | 7 | 3 |
|  | NSCLC* | 144 | 94 |
|  | Other Lung Cancer | 5 | 0 |
|  | Unknown Sub type | 9 | 3 |
|  | Normal | 0 | 0 |

*Small cell and non small cell lung cancer are denoted by the abbreviations SCLC and NSCLC, respectively.
